# Processing of Donor Human Milk: Update and Recommendations From the European Milk Bank Association (EMBA)

**DOI:** 10.3389/fped.2019.00049

**Published:** 2019-02-28

**Authors:** Guido E. Moro, Claude Billeaud, Buffin Rachel, Javier Calvo, Laura Cavallarin, Lukas Christen, Diana Escuder-Vieco, Antoni Gaya, David Lembo, Aleksandra Wesolowska, Sertac Arslanoglu, Debbie Barnett, Enrico Bertino, Clair-Yves Boquien, Corinna Gebauer, Anne Grovslien, Gillian A. Weaver, Jean-Charles Picaud

**Affiliations:** ^1^Associazione Italiana delle Banche del Latte Umano Donato, Milan, Italy; ^2^Neonatology Nutrition, Lactarium Bordeaux-Marmande, CIC Pédiatrique 1401 Children's Hospital, Bordeaux, France; ^3^Lactarium Auvergne Rhone Alpes, Hospices Civils de Lyon, Lyon, France; ^4^Fundació Banc Sang i Teixits de les Illes Balears, Palma de Mallorca, Spain; ^5^Institute of Sciences of Food Production, National Research Council, Turin, Italy; ^6^CARAG AG, Baar, Switzerland; ^7^Banco Regional de Leche Materna, Hospital Universitario 12 de Octubre, Madrid, Spain; ^8^Laboratory of Molecular Virology, Department of Clinical and Biological Sciences, University of Turin, Turin, Italy; ^9^Department of Neonatology, Faculty of Health Science, Medical University of Warsaw, Warsaw, Poland; ^10^Division of Neonatology, Department of Pediatrics, Istanbul Medeniyet University, Istanbul, Turkey; ^11^Greater Glasgow and Cycle Donor Milk Bank, Royal Hospital for Sick Children, Glasgow, United Kingdom; ^12^Neonatal Unit of Turin University, City of Health and Science, Turin, Italy; ^13^PhAN, Institut National de la Recherche Agronomique, Université de Nantes, CRNH-Ouest, Nantes, France; ^14^Abteilung Neonatologie Klinik und Poliklinik für Kinder und Jugendliche, Leipzig, Germany; ^15^Breast Milk Bank, Oslo University Hospital, Oslo, Norway; ^16^The Milk Bank, Imperial College Healthcare NHS Trust, London, United Kingdom; ^17^CarMeN Unit, INSERM U1397, Claude Bernard University Lyon 1, Lyon, France

**Keywords:** processing of human milk, donor human milk, human milk, human milk bank, preterm infants, infant nutrition

## Abstract

**Background:** A mother's own milk (MOM) is the gold standard for the feeding and nutrition of preterm and full term infants. When MOM is not available or there is not enough, donor human milk (DHM) should be used. Milk delivered to Human Milk Banks (HMBs) should be pasteurized to inactivate viral and bacterial agents. Currently, a pasteurization process at 62.5°C for 30 min (Holder pasteurization, HoP) is recommended in all international HMBs guidelines.

**State of the art:** It is known that HoP affects some of the nutritional and biological components of human milk. Studies have demonstrated that temperature cycle in HoP is not always controlled or calibrated. A better check of these parameters in the pasteurizers on the market today may contribute to an improvement of the quality of HM, still maintaining some of the negative effects of the heat treatment of human milk. So, food industry, and dairy industry in particular, are evaluating innovative methodologies alternative to HoP to better preserve the nutritional and biological properties of fresh human milk, while assuring at least the same microbiological safety of HoP. The most studied processing techniques include High-Temperature-Short-Time (HTST) pasteurization, High Pressure Processing (HPP), and Ultraviolet-C (UV-C) irradiation. HTST is a thermal process in which milk is forced between plates or pipes that are heated on the outside by hot water at a temperature of 72°C for 5–15 s. HPP is a non-thermal processing method that can be applied to solid and liquid foods. This technology inactivates pathogenic microorganisms by applying a high hydrostatic pressure (usually 300–800 MPa) during short-term treatments (<5–10 min). UV irradiation utilizes short-wavelength ultraviolet radiation in the UV-C region (200–280 nm), which is harmful to microorganisms. It is effective in destroying the nucleic acids in these organisms, so that their DNA is disrupted by UV radiation.

**Aim:** The aim of this paper is to present the EMBA recommendations on processing of HM, based on the most recent results obtained with these new technologies.

**Conclusions:** Although research on the most promising technologies that will represent an alternative to HoP (HTST, HPP, UV-C) in the future is progressing, it is now important to recognize that the consistency and quality assurance of the pasteurizers on the market today represent a fundamental component that was previously lacking in the Holder approach.

## Background

The increasing number of infants who are born extremely preterm and survive at birth and beyond, with a gestational age as low as 22 weeks, represents a new challenge for neonatal nutrition. In the last few decades, human milk (HM) has been identified as the normative standard for premature infant feeding and nutrition by health organizations and scientific societies (1–3). HM confers to these infants protection against necrotizing enterocolitis, sepsis and other infections, and severe retinopathy, decreases the risk of death, and improves their long-term neurocognitive development and cardiovascular health. In addition, the benefits of breast-feeding to promote psychological health and mother-infant bonding are well-known. A mother's own milk (MOM) is the first choice for premature infant feeding. When there is not sufficient MOM (a common occurrence in Neonatal Intensive care Units), donor human milk (DHM) obtained from well-established human milk banks (HMBs) is the best alternative. The WHO/UNICEF Joint Statement clearly indicates: “HMBs should be made available in appropriate situations” (4).

DHM delivered to HMBs should be pasteurized to inactivate viral and bacteriological agents (5). The ideal pasteurization process should consist of a rapid heating phase, followed by a phase in which the temperature is maintained constant, and a final rapid cooling phase. Currently, a pasteurization process performed at a temperature of 62.5°C for 30 min, which is known as the Holder pasteurization (HoP) method, is recommended in all international guidelines for the establishment and management of HMBs (5, 6). Pasteurized HM is known to retain many beneficial and protective components of fresh HM (5). However, it also affects some of the nutritional and biological properties of HM and eliminates the beneficial microbiota of fresh HM, thus resulting in the reduction of some bacteriostatic mechanisms that render milk more susceptible to postheating bacterial contamination, and decreases in its nutritional value (5).

Due to the present limitations of HoP in processing of HM, there is the need to evaluate alternative processing methods able to preserve better the bioactivity of a higher number of HM components in order to improve the nutritional and immunological quality of DHM.

New technologies are under study and the purpose of this manuscript is to evaluate the results obtained from the most promising ones.

## State of the Art and Future Trends

HM is a functional and dynamic biologic system: it provides nutrients, bioactive components and immune factors, and it promotes an adequate and healthy growth of newborn infants. Milk delivered to HMBs should be pasteurized to inactivate viral and bacterial agents, as well as a mother's own milk in specific clinical situations.

Currently, HoP is the most frequently studied and recommended method for the heat treatment of donor HM (5–8). A recent review has shown a significant variability in the data reported in scientific literature concerning the effects of HoP on the biological components of HM (9). Possible explanations for this variability could be the heterogeneity of the test protocols applied in the studies, the fact that HoP is often simulated in laboratories on small aliquots of milk rather than being performed with commercial instruments on the larger volumes of milk utilized inside the HMBs, and, last but not least, modern pasteurizers require significantly less time for heating and cooling than older ones, thus changing the kinetics of the thermal response for heat-sensitive compounds (9–12).

The loss of some biologically active components as a result of HoP, including immunological components, is the main limit to the spread of donor HM utilization in the feeding of preterm infants (10, 11).

The optimization of the biological and nutritional quality of DHM is considered, by the European Association of Human Milk Banks (EMBA), as a scientific and social priority. In order to investigate this aspect, the EMBA Board of Directors set up a Working Group (WG), that is, a dynamic network of scientists who perform research in the field of HMBs and HM treatment, and who operate in different European countries. This WG is aimed to evaluate old and new methodologies in order to determine their effects on the final quality of DHM delivered from HMBs. The objective is to obtain optimum levels of quality and safety of DHM from milk banks in Europe and to decrease the variability of HM, at least as far as the aspects related to the effects of heat treatment are concerned. Quality has been discerned as a powerful tool for the improvement of the well-being of premature infants. A better management of DHM in HMBs will improve the services for donor women (those who donate milk) and for the recipients (the newborn infants who receive it).

The main technologies taken into considerations by this WG are the following: low-temperature long-time pasteurization (LTLT), which has been evaluated by a French group in Lyon (RB and JCP); high-temperature short-time pasteurization (HTST), evaluated by two Italian groups located in Turin and Milan (EB, LC, DL, and GEM); high pressure processing (HPP), evaluated by a French group located in Bordeaux (CB and GD) and a Polish group located in Warsaw (AW); and ultraviolet (UV) irradiation, performed by the Spanish group located in Palma de Mallorca (JC and AG).

### Low-Temperature Long Time Pasteurization

At present, the most common practice utilized for the treatment of DHM is a low-temperature (62.5°C) long-time (30 min) pasteurization (LTLT), which is known as Holder pasteurization (HoP). HoP is recommended in all the international guidelines. Milk pasteurization with HoP is known to retain many of the beneficial and protective effects of HM, such as a reduction in NEC and sepsis, protection against bronchopulmonary dysplasia, and a lower rate of long-term complications, such as cardiovascular diseases and neurodevelopmental disabilities (3).

However, some significant concerns have arisen, related to the possible alterations of the nutritional and biological properties of DHM, as a result of the heat treatment. HoP produces a loss in the quantity and/or activity of some biologically functional milk components to varying degrees (9). Other nutritional and biological components, such as oligosaccharides, lactose, glucose, LCPUFAs, gangliosides, vitamins A, D, E, and B12, folic acid, some cytokines, and some growth factors are instead preserved (9).

Different devices have been produced to perform LTLT pasteurization. The most common heat source for pasteurization is hot water, but moving hot air has also been used in some other devices. In 2017, Buffin et al. showed that air pasteurizers have a very different pasteurization pattern from water pasteurizers (12). When the temperature recorded in the different bottles inside at pasteurizer's bath was measured, it was not homogenous, with a difference of 21.7 min between the first probe and the last probe reaching 62.5°C. Moreover, the plateau duration was on average 10 min longer in air pasteurizers than in water pasteurizers. Therefore, the exposure to temperature seems to be more prolonged in the former devices (12). In fact, air is a less effective thermal conductor than water. Its propulsion is uneven and leads to temperature inhomogeneity in pasteurizer. This phenomenon leads to the bottles undergoing a different treatment from each other, and it is difficult to provide adjustments to improve the process.

Water is the most homogeneous environment heat conducting source, and it is therefore the most widely used medium for HoP. Different devices exist on the market today, but not all of them are provided with a temperature control system. The duration of the heat treatment and the maximum temperature of milk exposure have been shown to be essential for the preservation of human milk. Evans et al. already showed, back in 1978, that the alteration of the immunological components of human milk began at a temperature of 60°C and became more significant at 65°C (13). This was later confirmed by Czank et al. who demonstrated a significant impact of the temperature and an early alteration starting at 58°C (14). The study also demonstrated the influence of the duration of pasteurization, with a loss of 1.6, 1.7, and 2.4%, respectively, for IgA, lysozyme and lactoferrin per each minute spent at a temperature of 62.5°C (14).

The recording of the temperature of milk in several bottles, by means of external probes, during the pasteurization cycle showed significant differences, in terms of temperature or the duration of exposure of HM, depending on the device. The increase in the temperature of milk is in fact fast up to 58°C, but the inertia of heating is then responsible for a slowing down of the rise in temperature up to 62.5°C. It has been demonstrated that HM immune components start to be damaged significantly from 58°C (14). The regulation of each pasteurizer is therefore crucial to minimize the exposure time responsible for the damage to HM. Buffin et al. reported that the difference in exposure above 58°C could be as much as 10 min longer, depending on the device. In addition, the average temperature of the plateau can vary by nearly 0.8°C. These effects are only visible when several calibrated probes are used (12, 15). It is important to note that the milk was shaken during the heat treatment in both types of tested pasteurizers (12).

A single recording probe in just one of the bottles does not allow either the whole pasteurizing process to be understood or deviances in the system to be detected. Since HoP is currently conducted at a relatively high temperature (62.5°C), it is important to control this temperature and the duration of exposure (12, 15). Furthermore, most of the studies that have evaluated or compared HoP with other techniques have not described the pasteurizer cycle precisely. The differences that exist between pasteurizers can be important and can have an important impact on the assessed components. This could explain the discrepancies that have been found between the different results in literature. These inconsistencies make it difficult to make a definitive statement on the effects of HoP. For these reasons, any future study on HoP should adopt a standardized approach to ensure consistency. However, it is important to recognize that where research on HoP is being carried out, consistency, and quality assurance adds a necessary component that was previously lacking in the approach.

Therefore, a routine recording of the milk temperature in one bottle located in the middle of a bath is important to control each pasteurization cycle. This probe is not present in all the pasteurizers available on the market today, and is present even less in a simple water bath. Each pasteurizer should be made to undergo regular quality controls, performed by each HMB using several external probes. Some criteria have recently been proposed (Table 1) (12, 15). Since 2016, such quality controls have been performed regularly, at least once a year, in the 36 French HMBs. Manufacturers should provide these criteria when they propose qualified pasteurizers to HMBs.

**Table 1:** Criteria for qualification of human milk pasteurizers (12, 14, 15).

Measurement by calibrated temperature probes Independent of the pasteurizer Regular distribution of the probes inside the pasteurizer One probe for 8–10 bottles Qualification repeated once a year and after major intervention, and performed on three pasteurization cycles Temperature of the plateau as close as possible to 62.5^°^C and below 64^°^C Duration of the plateau as close as possible to 30 min and <35 min (time calculated when all probes have reached 62.5^°^C) Exposition time over 58^°^C <50 min for each probe Exposition time from 62.5 to 6^°^C ≤ 1 h

This qualification has two purposes:

- The first is to highlight a dysregulation of the pasteurizer;
- The second, which is based on the results, is to require the manufacturer to adjust and optimize the regulation of the pasteurizer in order to minimize the temperature plateau range, the duration of the pasteurization plateau, and to ensure cycle accuracy and repeatability.

Moreover, since HoP is the most frequently used technique, it should be considered, as part of the optimization, whether a value of 62.5°C is the best temperature for the treatment of human milk. Czank et al. have shown the effectiveness of pasteurization on bacteria at temperatures above 57°C. However, it is known that Cytomegalovirus persists at this temperature, but it could be useful to test intermediate temperatures, such as 60°C and / or shorter exposure times (14).

Finally, it should be kept in mind that the heating phase should be followed immediately by a rapid chilling phase to 4°C to minimize the additional time during which the milk is exposed to the high water bath temperatures and to reduce the further destruction of heat labile components. This thermic shock could also prevent aerobic spore-forming bacteria from multiplying.

As long as HoP remains the main technique utilized in HMBs, it should be made as optimal as possible, with quality assurance being obtained through checks and calibration. When comparing HoP with new techniques, it should be ascertained that HoP is performed correctly, and the comparison should be made in conditions as close as possible to the routine daily practices in the HMBs.

### High-Temperature Short-Time (HTST) Pasteurization

HTST was the first non-HoP technique tested to improve the nutritional and immunological quality of milk, and it was first established in the dairy industry in the 1930s (16). HTST is usually performed by heating thin layers of milk in continuous flow systems at 72°C for 15 s. This technology has been applied to the treatment of DHM with promising results (9). Immunological components, and in particular immunoglobulins (Igs), are known to be affected by HoP (17–19), and have often been targeted as qualitative/functional parameters in studies on alternative HM pasteurization technologies. Goldsmith et al. were the first to test HTST pasteurization on HM using a stainless steel laboratory capillary heat exchanger (20). They reported comparable degradation after HTST and HoP for Igs and lactoferrin. The retention of HM Igs was found to decrease as the temperature and holding time increased. Therefore, the search for an optimal compromise between microbiological safety and biological quality should be made considering the pasteurization equipment and the working conditions. Data regarding the effect of HTST pasteurization on lactoferrin and lysozyme concentrations and activities are sometimes divergent, due to the fact that different methods were used to apply the HTST technology in the different studies.

Overall, it has emerged that HTST performed at a laboratory scale or pilot scale is at least equivalent to HoP in ensuring HM microbiological safety, but is better at preserving the HM antioxidant potential, lactoferrin content and structure, B and C vitamins, and some cytokines.

Two HTST pasteurizers have recently been specifically designed and validated for human milk processing (21, 22).

In the first study, a new small-scale, continuous-flow, HTST pasteurizer was designed to treat HM. The efficacy of the new HTST device was assessed on inoculated *Listeria monocytogenes, Staphylococcus aurous, and Chronobacter sakazakii*, as well as on raw human milk bacteria. The biological quality of the milk was assessed after HTST pasteurization and compared with a standard HoP, by determining the secretory IgA (sIgA) content, the protein profile, lysozyme and the Bile Salt Stimulated Lipase (BSSL) activities. No pathogen or bacterial growth was detected after HTST pasteurization with the new instrument. Changes in the protein profile were observed in the milk pasteurized with both processes. The sIgA content and BSSL activity were significantly higher in the milk pasteurized with the new device than in the same milk treated with the standard HoP. In conclusion, the new HTST apparatus was able to effectively pasteurize HM and showed a better retention of the sIgA content and a better BSSL activity (21). However, these results still have to be confirmed in HMB conditions.

Escuder-Vieco et al. described HTST equipment designed specifically for the continuous and adaptable (time-temperature combination) processing of DHM, considering the specific needs of a human milk bank (22). Microbiological quality, the activity of the indicator enzymes and indices for thermal damage to HM were evaluated before and after HTST treatment using different temperature and time combinations and the results were compared with the results obtained after HoP (22). The HTST system had an accurate and simple operation mechanism, which allowed the pasteurization of variable amounts of DHM and reduced both the processing time and the labor force. HTST processing at 72°, for at least 10 s, effectively destroyed all the vegetative forms of the microorganisms that were initially present in the raw DHM. Alkaline phosphatase was completely destroyed after HTST processing at 72 and 75°, but γ-glutamil transpeptidase showed higher thermoresistance, thus indicating that this could be used as a quick, simple, and inexpensive test. The furosine concentrations in HTST-treated donor HM were lower than those obtained after HoP, and the lactulose content for HTST-treated DHM was below the detection limit of the analytical method (10 mg/L). The absence of lactulose and the small amount of furosine found in HTST-treated DHM indicated that a heat treatment with this new HTST equipment did not induce any significant heat damage to DHM. In addition, a higher retention of immunoglobulins, some hormones, BSSL activity and antioxidant capacity were found in HTST-treated DHM samples than in the samples treated by means of HoP (22).

### High Pressure Processing (HPP)

HPP is a well-known technique in the food industry, and it is considered a promising alternative to the thermal pasteurization of HM. HPP is a non-thermal processing method that can be applied to solid and liquid foods to provide microbiologically safe, nutritionally intact, and sensory high-quality products (23). This technique inactivates pathogenic microorganisms by applying high hydrostatic pressure (usually 400–800 MPa) through short-term treatments (<5–10 min) (24).

Viazis et al. were the first to point out the retention of nutrients and the bioactivity and microbial safety of pascalized HM (25, 26).

Other researchers have shown that HM activity after processed over a 300 to 650 MPa HPP range is similar to heat-treated milk. IgA, IgM, IgG, lysozyme, lactoferrin, cytokines (EGF, TGF-β1 and TGF-β2, IL-6, IL-8, TNF-α, IL-12, IL-17, and IFN-γ) α- and δ-tocopherol, and free nucleotide monophosphates are partly preserved (27–32).

The destruction *of Listeria monocytogenes, Eschericha coli, Staphylococcus aurous, and Salmonella spp*, within the 300–400 MPa pressure range, is comparable with the microbiological purity obtained after thermal pasteurization (25, 28, 33).

Moreover, recently obtained results suggest that anHPP treatment, at pressures below 600 MPa for 15 min, allows the antirotaviral activity to be retained (34).

This technique respects the sensorial and nutritional properties of food better than HoP, because of the absence of a heat treatment (35, 36). As far as the safety and taste satisfaction of donor milk recipients are concerned, the profile of the volatile milk components has been examined after processing. Generally, the change in the sensory quality of human milk after a high-pressure treatment has been found to be less than that caused by HoP (35, 36).

It should be taken into consideration that a change in the lipid fraction may take place as a result of HPP. Milk fat is distributed as globules in colloidal fluid produced by the mammary gland. Any physical factor that is able to influence the stabilization of this component, either pressurization or a warm temperature, causes a decreased fat globule size, which is defined as a homogenization (37).

Exposure to pressures below 600 MPa has not been found to influence the content or composition of the lipid fraction of HM. However, increasing pressure above this limit might result in undesirable changes in the content of selected fatty acids in human milk. A risk of lipid oxidation products in HM after processing has been reported (38, 39).

The HPP technique seems to offer clear advantages over HoP: it results in an improved nutritional quality product; it is faster and perhaps more convenient than HoP; it can be applied to frozen milk samples and it can be used on samples of variable size.

A French team from Bordeaux and a Polish team from Warsaw, with representatives from the EMBA Working Group (CB and AW), have evaluated this technology with positive results. HPP seems to be able to better maintain some milk proteins (HGF, lactoferrin, IgG), and to preserve active hormones (leptine, adiponectine, insulin, erythropoietin) and enzymes (lipase) (EMBA International Conference on Donor Human Milk, Glasgow, October 5–6th, 2017). Until recently, it was considered that vegetative cells are more effectively destroyed by HPP than endosporic forms. Billaud and Demazeau have recently optimized the operational parameters (pressure, rate, decompression, and application mode) and this has allowed the inactivation of *B. cereus* spores. Under these conditions, the activity of certain important human milk biological components, such as lipase activity and immune proteins, is maintained. These results were obtained with a pressure of 350 MPa (40).

**Figure 1:**
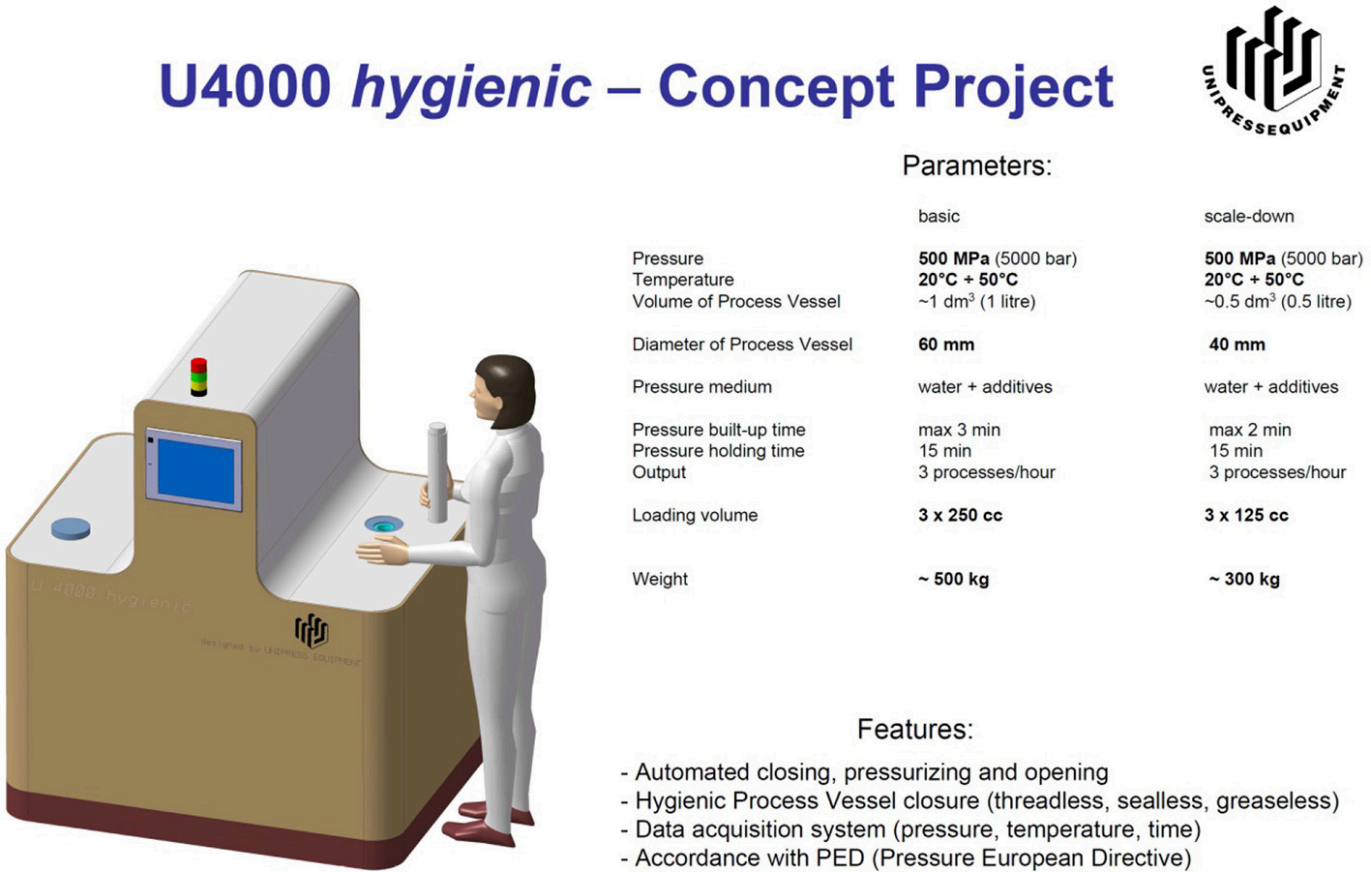
Prototype and parameters utilized for high pressure processing of human milk.

The main obstacle to the use of HPP in human milk treatment, is the scaling down of the equipment and the investment and operating costs. It has been calculated on the basis of a cost consequence analysis conducted with a regional model of human milk banking operating in Poland, that the cost of pascalized donor milk will be 130% higher than milk treated by means of Holder (unpublished data). However, there are some small and medium-size enterprises in Poland that are interested in investing in the human milk bank market. The prototype equipment for human milk pascalization has already been described, and the next step will be to obtain the money to construct and validate the device (Figure 1).

At present, only prototypes of these HPP devices exist, and this technique still has to be tested under HMB conditions.

### Ultraviolet-C Irradiation

Ultraviolet (UV) irradiation utilizes short-wavelength ultraviolet radiation in the UV-C region (200–280 nm), which is harmful to microorganisms. It is effective in destroying the nucleic acids in these organisms, so that their DNA is disrupted by the UV radiation, leaving them unable to perform vital cellular functions. The greater the exposure to UV rays, the better the result, and this ensures a complete destruction of all the microorganisms (1, 3). UV light only penetrates food materials by several millimeters, depending on the optical properties of the product. Ultraviolet light penetrates the cells, but does not alter the food that is being treated. The color and/or turbidity of the liquid influences its optical absorption coefficient. UV light cannot penetrate milk or other cloudy foods, like other opaque foods. As a consequence, these substances must be presented to the system as a thin layer, and this constitutes a concern when large volumes of donor HM in HMBs are being treated daily (41, 42).

Some preliminary reports have shown that UV irradiation is able to produce a reduction of 5 log 10 in the exogenously-added bacteria in HM, without affecting the lipase activity (43). The concentrations of lactoferrin, lysozyme and immunoglobulin A (IgA) have been described as basically being unaltered (44), and it has also recently been reported that ultraviolet -C radiation is able to inactivate cytomegalovirus in HM under the correct conditions (45).

**Figure 2:**
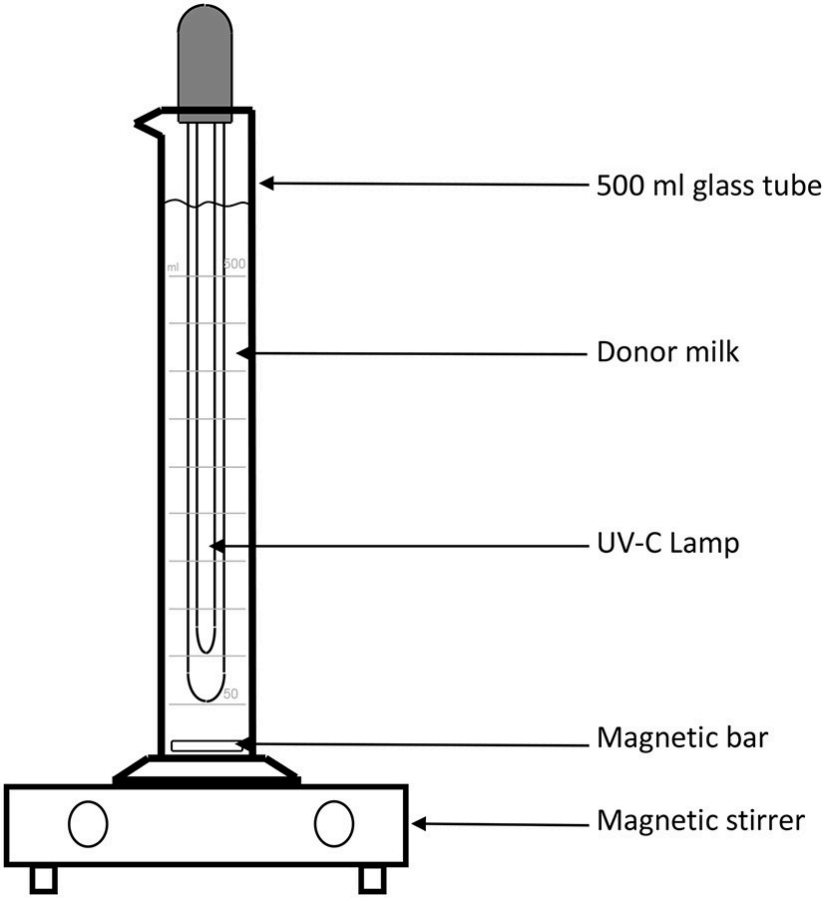
Schematic representation of the device used to treat donor human milk with UV-irradiation.

The main challenge to testing this methodology is the lack of appropriate equipment in the human milk bank context. In order to further analyze the potential application of UV-C irradiation in this context, a Spanish group from Palma de Mallorca (JC and AG), has designed an instrument in which milk is kept in motion, through the use of a magnetic stirrer bar, which creates a low velocity, laminar flow vortex, thus transporting and overcoming the low penetrance of UV irradiation. Figure 2 demonstrates the instrument, which allows 500 ml of milk to be processed: it consists of a graduated cylinder glass tube in which a 26 cm long UV-C lamp with 8 w power has been introduced, so that 10 min of treatment equals 9,600 Joules/Liter (LIT-06; Instrumentación Científico Técnica S.L., La Rioja, Spain). In this treatment, the milk is kept at room temperature and agitated with a magnetic bar and a stirrer at 200 rpm.

With this device, Calvo and Gaya tested five different 500 ml batches of DHM that had been discarded during routine processing in their HMB due to the presence of high levels of contamination. Of the five analyzed samples, two presented >10^5^ CFU/ml and three >10^6^ CFU/ml. In all cases, the contamination was due to a mixed flora, including gram-negative bacteria. A sample was taken at different times after the treatment was started (0, 15, 30, 45, and 60 min) and the number of CFU was quantified by means of conventional microbiological techniques. These experiments showed that, after 30 min of treatment, the amount of CFU/ml was reduced by five orders of magnitude (log10) in all cases. The fact that the used samples were representative of the DHM samples usually treated in a HMB is of particular relevance.

Bacillus is a bacteria genus that is frequently found in DHM, and it represents a special concern for HMBs, as these bacteria are capable of producing heat-resistant toxins and forming spores that are resistant to pasteurization (46, 47). In the HMB of Palma de Mallorca, about 10% of the milk is generally discarded due to the presence of *Bacillus sp*. In order to test the susceptibility of *Bacillus sp* to the UV treatment, the researchers used two different batches of donor milk that had been discarded as a result of contamination with *Bacillus* sp., 3,000 CFU/ml in both cases. The results showed that after 45 min of treatment, the *Bacillus sp*. were eliminated.

The Spanish group then evaluated the effect on the biological components of HM. One of the main biological components is IgA, which constitutes 90% of all the immunoglobulins present in colostrum and HM. Its importance lies not only in its concentration, but also in its biological activity (48). It has been pointed out that pasteurization affects IgA levels to different extents, depending on the pasteurization temperature (14). In the case of HoP, a clear decrease in IgA concentration was observed, although there were large discrepancies in the range of reduction, from 20 to 60% (9). The results of this group have shown that, after testing seven different batches of DHM, the IgA levels measured by conventional nephelometry techniques, were 96% of the pre-treatment levels, and in five samples, a 100% activity was preserved.

From these results, it can be concluded that the treatment with UV-C light has a number of features that make it a good candidate as an alternative to HoP. In addition to providing a better protection of the biological components than other methods, it is also capable of producing an at least 5-log10 decrease in the number of bacteria (including *Bacillus* sp) present in DHM. Furthermore, the ability of UV-C radiation to eliminate active forms of Cytomegalovirus in HM has also been demonstrated (45). Unfortunately, until now, there is neither a device nor even a prototype that would enable the use of this technology in an HMB setting.

### EMBA Working Group Recommendations

One important aspect that should be considered when evaluating the processing of human milk is the viral inactivation effect of the new methodologies.

The ability of LTLT pasteurization to inactivate viral pathogens is well-known. The list of human viruses inactivated by HoP includes pathogens for which transmission through breastfeeding has been conclusively demonstrated (i.e., the human immunodeficiency virus, human T-cell lymphotrofic virus, cytomegalovirus), and viruses that can be transmitted via breast milk, such as human papillomavirus, Zikavirus, Ebola and the Marbourg virus (49–55).

On the other hand, virus inactivation still has to be carefully evaluated for each alternative technique and device designed to treat breast milk. This is an important issue for future research.

We can state that fundamental knowledge of new technologies of HM processing is still lacking. Their effects on safety and bioactive components of HM need further evaluation. Table 2 presents the “state of the art” at the moment, explaining advantages, and disadvantages of the processing techniques described in this paper.

**Table 2:** Advantages and disadvantages of the processing techniques described in this paper.

**Processing Technique**	**Advantages**	**Disadvantages**
Low-Temperature Long-Time Pasteurization (LTLT), known as Holder Pasteurization (HoP)	- Best known methodology - Recommended in all international guidelines for the constitution of Human Milk Banks - Well-established antimicrobial and antiviral activity - Retention of many beneficial and protective effects of human milk	- Reduction/disruption of important nutritional and immunological factors of human milk - Ineffective against bacterial spores (*Bacillus cereus*) - Need of regular requalification of the pasteurizer
High-Temperature Short-Time Pasteurization (HTST Pasteurization)	- Utilized in dairy industry since 1930s - Less thermal stress (processing time in seconds and not in minutes) - Better retention of sIgA and lipase activity in comparison to HoP - Smaller loss in antioxidant potential than HoP	- Prototypes have been used for comparative studies - No device available on the market today - Ineffective against bacterial spores (*Bacillus cereus)*
High Pressure Processing (HPP)	- No thermal stress (processing at low temperature) - Better retention of some important biological components (lipase, lysozyme, lactoferrin, IgA) in comparison to HoP - Inactivation of bacterial spores - Higher microbial safety	- Antiviral activity needs a more deep evaluation - Investment and operating costs are significantly higher than a conventional pasteurizer - Scaling down of the equipment represents a practical problem - Dimensions and weight of the apparatus make difficult the placing in human milk banks
Ultraviolet-C irradiation (UV irradiation)	- Emerging food preservation technique that retains higher quantities of bioactive components - Better retention of IgA in comparison to HoP - Effective on elimination of *Bacillus cereus* spores	- Application of UV-C technology is difficult in human milk - Only few preliminary reports are available - Antiviral activity has to be evaluated - Lack of appropriate equipment in a human milk bank setting

On the basis of evidence taken from the literature and on the personal experience of its members, the Working Group on the Processing of Human Milk makes the following recommendations:

When testing new technologies, the following requirements should be fulfilled:

- The equipment should be described precisely
- The control of the equipment and repeatability of the process should be demonstrated
- The process parameters should be recorded
- Tests should not be performed only at a lab scale, but also in an HMB environment

The final aim of HM processing performed with new technologies should be:

- To improve the preservation of the nutritional and bioactive components of raw HM (in order to at least ensure the same microbiological safety as HoP)
- To improve the microbiological safety of treated DHM, taking into account the inactivation of spores, even though this aspect is not at present considered in all the guidelines that regulate the activity of HMBs
- To inactivate the viral effect on human viruses for which transmission through breastfeeding has been demonstrated
- Easy placement of the new plant in HMBs
- Low cost, in order to overcome the problem of the limited financial resources of the majority of HMBs.

**Figure 3:**
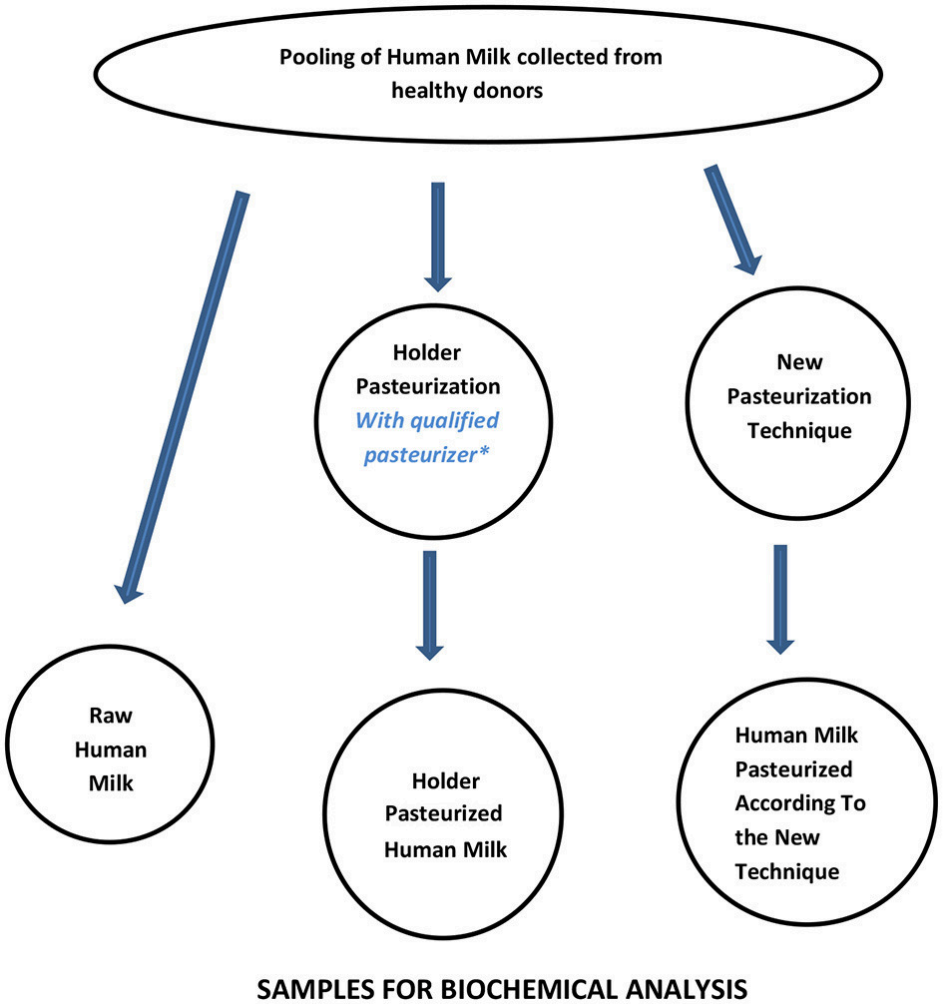
Workflow for assessing the performance of new pasteurization technologies for human milk. *For the qualification of the Holder pasteurizer, see Buffin et al. (12).

A workflow that can be considered suitable to assess the basic performance of new pasteurization technologies for HM is shown in Figure 3 and Table 3. Since Holder pasteurization is not efficient in eradicating spore-forming bacteria, this parameter has not been included in the validation targets. However, any new pasteurization system that could prove to be efficient against spore-forming bacteria (while maintaining all the other aforementioned characteristics) would represent a great advantage for the improvement of HM safety.

**Table 3:** Parameters to evaluate for validation of new pasteurization technologies.

**BIOCHEMICAL QUALITY**			
**Parameter**	**Unit**		**Method**
sIgA	ng/ml		Giribaldi et al. (21)
BSSL activity	μmol/min/ml		Giribaldi et al. (21)
Lysozyme activity	U/μl		Giribaldi et al. (21)
**MICROBIOLOGICAL QUALITY^§^**			
**Inoculated bacteria**	**Initial loads in raw milk (CFU/mL)**	**Final loads in pasteurized milk (CFU/ml)**	**Method**
*Listeria monocytogenes*	1.2 × 10^6^	Absent in 25 ml	EN/ISO 11290, 1996 (21)^°^
*Staphylococcus aureus*	3.0 × 10^6^	<100	EN/ISO 6888, 1999 (21)^°^
*Chronobacter sakazakii*	1.6 × 10^6^	Absent in 10 ml	AFNOR V08-054, 2009 (21)^°^
CMV		Absent	Hamprecht et al. (51)^°^
HIV		Absent	Giribaldi et al. (21)^°^

§ *For the design of the microbiological challenge test, see Giribaldi et al. (21)*.

° *Or equivalent methods*.

## Conclusions

This paper presents the recommendations of the EMBA Working Group on the “Processing of HM.” Although research on the most promising technologies, which will represent a reasonable alternative to HoP in the future (HTST, HPP, UV-C) is progressing, at the moment it is important to recognize that the consistency and quality assurance of the pasteurizers currently available on the market today represent a fundamental approach that was previously lacking in HoP practice.

EMBA recognizes that HoP is at present the safest compromise for the treatment of DHM; however, further studies are needed to improve this technology in order to minimize its effects on the biological components of HM. The new technologies evaluated and studied by the Working Group are being developed rapidly, and EMBA recommends that the final aim of these technologies should be an improved preservation of the nutritional and bioactive components of raw human milk, while assuring microbiological safety of the product, at least at the same level as optimized HoP.

## Author Contributions

GEM wrote the manuscript. CB, BR, JC, LaC, LuC, DE-V, AGa, DL, and AW are components of the EMBA Working Group on Processing of Donor Human Milk and contributed to the content of this manuscript. SA, EB, AGr, DB, C-YB, CG, GW, and J-CP are components of the Board of Directors of EMBA and made comments and gave suggestions for the final preparation of the manuscript.

### Conflict of Interest Statement

LuC is employed by Carag AG, Switzerland. However, Carag AG did not offer any financial support for this paper. The remaining authors declare that the research was conducted in the absence of any commercial or financial relationships that could be construed as a potential conflict of interest. The reviewer MG declared a past co-authorship with the authors JC and AGa to the handling editor.
